# The Heparanase Regulatory Network in Health and Disease

**DOI:** 10.3390/ijms222011096

**Published:** 2021-10-14

**Authors:** Alyce J. Mayfosh, Tien K. Nguyen, Mark D. Hulett

**Affiliations:** Department of Biochemistry and Genetics, La Trobe Institute for Molecular Science, La Trobe University, Melbourne, VIC 3083, Australia; a.mayfosh@latrobe.edu.au (A.J.M.); 19573282@students.latrobe.edu.au (T.K.N.)

**Keywords:** heparanase, heparan sulfate, extracellular matrix, cytokine, growth factor, gene regulation

## Abstract

The extracellular matrix (ECM) is a structural framework that has many important physiological functions which include maintaining tissue structure and integrity, serving as a barrier to invading pathogens, and acting as a reservoir for bioactive molecules. This cellular scaffold is made up of various types of macromolecules including heparan sulfate proteoglycans (HSPGs). HSPGs comprise a protein core linked to the complex glycosaminoglycan heparan sulfate (HS), the remodeling of which is important for many physiological processes such as wound healing as well as pathological processes including cancer metastasis. Turnover of HS is tightly regulated by a single enzyme capable of cleaving HS side chains: heparanase. Heparanase upregulation has been identified in many inflammatory diseases including atherosclerosis, fibrosis, and cancer, where it has been shown to play multiple roles in processes such as epithelial-mesenchymal transition, angiogenesis, and cancer metastasis. Heparanase expression and activity are tightly regulated. Understanding the regulation of heparanase and its downstream targets is attractive for the development of treatments for these diseases. This review provides a comprehensive overview of the regulators of heparanase as well as the enzyme’s downstream gene and protein targets, and implications for the development of new therapeutic strategies.

## 1. Introduction

The extracellular matrix (ECM) is a complex three-dimensional structural network comprised of proteins and polysaccharides that surround cells and tissues in multicellular organisms. This extracellular architecture is responsible for offering structural support and integrity to tissues and provides protection from invading cells and pathogens. It also has roles in many cellular processes, including cell survival, growth, migration, and differentiation [1]. Key components of the ECM include proteoglycans and fibrous proteins such as collagen, elastin, fibronectin, and laminin. Of particular interest to this review are the heparan sulfate proteoglycans (HSPGs).

The HSPGs are comprised of a protein core with covalently linked side chains of the variably sulfated glycosaminoglycan heparan sulfate (HS). HSPGs are found within the ECM (agrin, perlecan, and type XVIII collagen), bound to the cell membrane (syndecans and glypicans), or within secretory vesicles (serglycin) [2,3]. They can also be found in the nucleus [4].

There are many proteins that bind HS (Table 1). Indeed, over 400 human proteins have been shown to bind HS or the structurally related heparin [5] (where heparin-binding likely predicts HS-binding abilities). Many of these binding proteins have been confirmed by proteomic, surface plasmon resonance, and column chromatographic methods. These proteins include growth factors, e.g., fibroblast growth factor (FGF); cytokines, e.g., monocyte chemoattractant protein-1 (MCP-1); and other ECM components, e.g., collagen. HS-binding molecules either interact through a specific HS-binding sequence motif, e.g., FGF [6] or in a nonspecific charge-dependent manner, e.g., fibronectin [7,8]. By binding HS, these proteins are sequestered within the matrix. Many also require HS for activity; for example, the formation of many chemokine gradients requires HS to facilitate chemokine oligomerization [9].

The abundance of HS on the cell surface and its importance in several pathways led to the discovery that HS acts as a co-receptor for several signaling receptors. These include the FGF receptor (FGFR) where cell surface HS is required for activation of the receptor [10] and vascular endothelial growth factor receptor (VEGFR) where HS can activate VEGFR in trans- from neighboring cells [11]. HS expressed on the surface of endothelial cells also acts as an adhesion receptor for migrating lymphocytes [12]. Given the diverse roles of HS in normal physiology and disease, its regulation and turnover are important to understand. In mammals, the turnover of HS and therefore ECM homeostasis is regulated by one enzyme: heparanase.

Heparanase is a member of the glucuronidase family and recognizes HS polysaccharide chains at sites of high sulfation. It catalyzes the hydrolysis of the β-linkage joining glucuronic acid and N-acetylglucosamine residues in HS chains, generating polysaccharide fragments of 10–20 units long [48]. Heparanase has several roles in physiological functions including wound healing [49] and leukocyte trafficking [50,51,52,53]. It also plays many roles in a number of different disease settings such as cancer and inflammatory diseases, where heparanase expression is upregulated and contributes to disease progression, making it an important enzyme to study.

Heparanase expression is regulated by several factors, such as cytokines, growth factors, and metabolites. In turn, heparanase can modulate the expression of several other genes and regulate the activity and bioavailability of various proteins and molecules. A description of this network in its entirety, including in all physiological and disease settings, has not yet been described in this form. Here we present an overview of this heparanase network as well as discuss how these links impact disease and what this understanding will mean for linking heparanase to disease diagnosis and treatment.

## 2. Regulation of Heparanase Expression

### 2.1. Heparanase Expression and Tissue Distribution

The human heparanase gene is located at chromosome 4q21.23 and spans 40 kb. The human, mouse, and rat heparanase genes are highly conserved, with the human and animal heparanase amino acid sequences sharing at least 80% identity. Under normal physiological conditions, the heparanase promoter is silenced by methylation [54,55]. Certain single nucleotide polymorphisms (SNPs) arising within the heparanase gene are associated with altered heparanase gene expression [56]. These same SNPs are also associated with heparanase mRNA expression in hematological malignancies [57].

The physiological expression of human heparanase was first reported in only the placenta and immune organs including the spleen, lymph node, peripheral blood, bone marrow, and fetal liver [58]. High expression has now been widely confirmed in immune cells, as well as observed in the esophagus, lung, heart muscle, keratinocytes, endothelial cells, and placental trophoblasts [59]. Recent advances in cell separation and RNAseq have allowed for detection of heparanase expression with increased sensitivity (less than 5 transcripts per million) in other human tissues including in the brain, endocrine organs, and the digestive tract [60] (Data available from https://www.proteinatlas.org/ENSG00000173083-HPSE/tissue, accessed on 16 June 2021).

**Figure 1:**
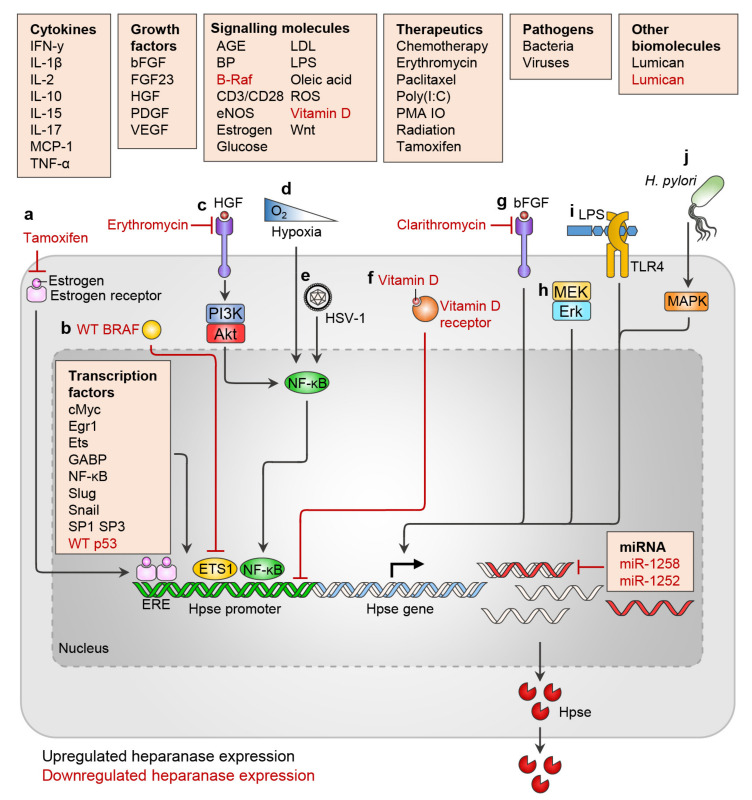
Regulators of heparanase expression. Heparanase expression is positively and negatively regulated by a number of cytokines, growth factors, signaling molecules, therapeutics, pathogens, transcription factors, and miRNA. (**a**) Estrogen binding to the estrogen receptor allows binding to the estrogen response element within the heparanase promoter and heparanase upregulation. (**b**) Wild-type BRAF inhibits heparanase expression by directly repressing ETS1, a transcription factor known to promote heparanase expression. (**c**) HGF, via the PI3K/Akt pathway, activates NF-κB to induce heparanase expression. Erythromycin inhibits HGF (and PDGF)-induced heparanase upregulation. (**d**) Hypoxia, and (**e**) HSV-1 upregulate heparanase via NF-κB. (**f**) Vitamin D activates the vitamin D receptor which directly binds and inhibits the heparanase promoter. (**g**) bFGF upregulation of heparanase can be inhibited with clarithromycin. (**h**) Activation of the MEK Erk pathway upregulates heparanase. (**i**) LPS binding to TLR4 upregulates heparanase. (**j**) *H. pylori* infection upregulates heparanase via MAPK. ERE, estrogen response element; Hpse, heparanase.

During normal cellular processes, heparanase expression can be upregulated in response to various stimuli, for example, upon immune cell activation [50,61,62,63]. Expression of heparanase is also dysregulated in many disease settings, such as its upregulation in cancer [64]. Heparanase gene expression during physiological and pathological processes is modulated by several transcription factors, miRNAs, cytokines, growth factors, and other signaling molecules. As well as these host factors, bacteria, viruses, and certain therapeutics have also been shown to alter heparanase expression (Figure 1). These regulatory factors are summarised in Table 2.

### 2.2. Transcription Factors

Wild-type p53 is a master regulator of normal cell cycle and apoptotic processes [128]. During cellular homeostasis, heparanase gene expression is suppressed by wild-type p53 via direct binding to the heparanase promoter [77]. Thus, the mutation of p53 that can occur during oncogenesis results in aberrant heparanase expression. As well as a lack of repression, heparanase expression can be actively upregulated. Through cloning and sequencing of the heparanase promoter, the transcription factors GA-binding protein (GABP), specificity protein 1 (Sp1), and Sp3 were found to directly upregulate heparanase gene expression [70]. Early growth response 1 (EGR1) was later shown to also positively regulate heparanase gene expression through direct activation of the heparanase promoter [61,62,66,67]. Finally, NF-κB, a potent transcription factor downstream of many signaling pathways, can also increase heparanase expression in tumor cells [72,74,75,76].

### 2.3. miRNA

Micro RNAs (miRNAs) are emerging as important regulators of tumorigenesis given they regulate hundreds of mRNAs and are widely dysregulated in cancer [129]. In metastatic breast cancer cells, the miRNA miR-1258 was found to suppress heparanase expression and subsequently control tumor invasion and metastasis [79]. Patient tissues of invasive ductal carcinomas also exhibited lower levels of miR-1258 and higher heparanase expression relative to matched normal mammary gland tissue [79]. Another miRNA, miR-1252-5p, was also recently identified to regulate heparanase expression in multiple myeloma [80]. Since miRNAs show potential as directed therapeutics, miR-1258 may be a prospective candidate for treatment of heparanase-mediated metastatic cancer.

### 2.4. Cytokines

Heparanase plays several key roles during inflammation, including immune cell migration and cell signaling [130]. Thus, it is not surprising that several inflammatory cytokines have been shown to upregulate heparanase expression. These include interferon-γ (IFN-γ), interleukin (IL)-1β, IL-2, IL-15, IL-17, MCP-1 and tumor necrosis factor-α (TNF-α) [50,81,82,85,87,88,104,131]. It remains unclear how several of these cytokines upregulate heparanase expression, though it is likely that the heparanase gene is a downstream target of these cytokine signaling pathways. However, for cytokines in which the mechanism has been explored, it appears that the mechanisms may differ in different settings. One study found that heparanase upregulation in TNF-α treated endothelial cells was independent of NF-κB, PI-3K, MAP kinase, and c-Jun kinase, but was dependent on caspase 8 [82]. In contrast, another study found that canonical NF-κB signaling was required for TNF-α induced heparanase upregulation in endothelial cells [73]. Another study to show TNF-α induction of heparanase (during colitis-associated tumorigenesis) proposed that since TNF-α also induced upregulation of EGR1 [132,133] that TNF-α induced heparanase expression via activation of EGR1, although this is yet to be confirmed.

There are still gaps in our understanding of how these cytokines upregulate heparanase. Defining the mechanisms of cytokine-mediated heparanase upregulation and their contribution in different physiological and disease settings is required to fully understand the relationship between cytokine signaling and heparanase function. Despite our gaps in understanding of how cytokines upregulate heparanase, there are clearly multiple mechanisms at play during inflammatory responses. This multifaceted upregulation of heparanase likely ensures its robust expression and thus contributes to both normal immune responses and inflammatory disease pathologies.

### 2.5. Growth Factors

Growth factors can also regulate heparanase expression. Of these, VEGF was shown to act differentially depending on the setting: reducing heparanase expression in endothelial cells [82] and increasing heparanase expression in melanoma cells [92]. Hepatocyte growth factor (HGF) has also been shown to upregulate heparanase expression at the transcriptional level in lung and gastric cancer cells [89,90]. In contrast to TNF-α described above, HGF upregulated heparanase in gastric cancer cells through the PI3 kinase/Akt/NF-κB pathway [90]. A number of other growth factors—basic fibroblast growth factor (bFGF), FGF23, and platelet-derived growth factor—have also been shown to increase heparanase expression in cancer cells [89,91]. Thus, growth factors are another group of proteins that are central to regulating heparanase expression during physiological and pathological processes.

### 2.6. Hormones and Metabolites

Other signaling molecules can also regulate heparanase expression, including hormones, metabolites, and reactive oxygen species (ROS). Estrogen signaling has been shown to influence heparanase expression. Estrogen in breast cancer cells increases heparanase expression [100,102,103], and treatment of cholangiocarcinoma cells (bile duct cancer) with the estrogenic inducer 17β-estradiol upregulated heparanase mRNA [101]. Interestingly, estrogen stimulation of breast cancer cells at low concentrations induced higher expression levels of heparanase than high concentrations of estrogen [100]. During pregnancy, estrogen levels increase, which suggests pregnancy may protect against heparanase upregulation induced by low estrogen. Indeed, a clinical study found that the number of pregnancies correlates with a reduction in estrogen receptor-positive breast cancer risk [134]. Thus, it is possible that the induction of heparanase expression by low levels of estrogen in healthy breast tissue may contribute to the initiation of breast cancer.

The metabolites glucose and vitamin D also modulate heparanase expression [104,105,106,111]. Treatment of either podocytes in vitro or a rat model of proteinuria with vitamin D (1,25-D_3_) reduced heparanase mRNA expression [111]. Upon vitamin D binding, the vitamin D receptor directly bound to the heparanase promoter and blocked heparanase expression [111]. Furthermore, vitamin D deficient mice exhibited increased heparanase expression and activity [111]. This finding suggests that vitamin D may be a suitable treatment for proteinuria by targeting heparanase expression.

The induction of ROS has also been shown to regulate heparanase expression and secretion [106,109,110]. This suggests heparanase is regulated alongside other stress response genes. The mechanism of ROS-mediated heparanase upregulation has not been elucidated, however since ROS activates PI3K/AKT, MAPK signaling pathways, and NF-κB [135] which can upregulate heparanase, these pathways provide possible mechanisms of ROS-mediated heparanase upregulation.

### 2.7. Pathogens

An important role for heparanase during viral infection is emerging and has been recently reviewed [136,137]. Multiple viruses including Herpes Simplex Virus-1 (HSV-1), cytomegalovirus, and Dengue virus have been shown to hijack heparanase expression to facilitate infection (Table 2). By hijacking host pro-survival pathways and enabling viral egress, viruses exploit heparanase to their advantage. Other viruses, namely foot and mouth disease virus [138], respiratory syncytial virus [139], human papillomavirus [140], and hepatitis B virus [141], have been reported to require HS, the substrate of heparanase, for pathogenesis. This suggests they may also modulate heparanase expression to facilitate pathogenesis, but this is yet to be determined. Given the modulation of expression during infection, targeting heparanase during viral infection poses both diagnostic and therapeutic potential. The heparanase inhibitors heparin and the HS mimetic PI-88 were shown to inhibit poxvirus infection in vitro [142], but whether this was mediated via inhibiting heparanase activity was not directly tested. Further understanding of the modulation and role of heparanase during these infections is required to verify heparanase as a viable target.

Bacterial infection has also been shown to modulate heparanase expression. *Fusobacterium nucleatum,* which induces periodontal disease and can lead to oral carcinoma, was shown to increase heparanase expression upon infection in vitro [112]. *Streptococcus pneumoniae* infection in mice also increased heparanase protein levels [115]. Heparanase expression was also upregulated in mouse corneas following *Pseudomonas aeruginosa* (*P. aeruginosa*) infection [114], where the source of heparanase was from both infiltrating immune cells and the corneal epithelium. The gut pathogen *Helicobacter pylori* (*H. pylori*) also induced heparanase expression in gastric cancer cells and this was found to be dependent on MAPK signaling [113]. Furthermore, in a clinical cohort of gastric cancer patients with *H. pylori* infection, heparanase expression correlated with poor overall survival and relapse-free survival [113]. A negative correlation between heparanase expression and cancer survival has been shown many times previously [143,144,145]. In the context of chronic bacterial and viral infections that can contribute to tumorigenesis, heparanase expression during this inflammatory pre-tumorigenic phase is likely a driver of tumorigenesis. There are other bacterial pathogens such as *P. aeruginosa* and *Staphylococcus aureus* which also interact with and induce shedding of HSPGs to promote bacterial pathogenesis and are reviewed by Garcia and colleagues [146,147]. Given this, heparanase may also play a role in the pathogenesis of these bacterial infections. There may be many more bacterial species and viral strains which utilize heparanase for pathogenesis or induce a pro-inflammatory host response that drives heparanase expression, although this remains to be explored.

### 2.8. Therapies

Therapies such as chemotherapeutics, immune activators, and radiation have all been shown to modulate heparanase expression. The observation that heparanase can confer chemotherapeutic resistance in cancer cells (reviewed in [148]) led to the discovery that the chemotherapies bortezomib, carfilzomib, and doxorubicin can induce the upregulation of heparanase in vitro [76]. This upregulation of heparanase correlated with an increase in chemotherapeutic resistance through activation of the NF-κB pathway. This suggests that heparanase may be a potential target in overcoming chemoresistance. Indeed, later studies found that targeting heparanase can re-sensitize resistant tumor cells to chemotherapy and inhibit tumor cell growth in vitro and in vivo [149,150], presenting a promising approach to enhance chemotherapy response. One study identified in a colorectal cancer model that heparanase involvement in chemoresistance is 2-fold: (i) heparanase induces syndecan-1 shedding directly and (ii) heparanase induces upregulation of matrix metalloprotease-9 (MMP-9), which induces the binding of heparin-binding epithelial growth factor-like factor (HB-EGF) to epidermal growth factor (EGF) receptor (EGFR) and downstream MEK ERK signaling, leading to 5-Fluorouracil resistance [151]. These findings explain why tumor cells upregulate heparanase upon chemotherapy treatment and validate the use of heparanase as a chemotherapy-sensitizing target.

Given the role of heparanase in leukocyte functions, it is not surprising that compounds that modulate immune activation also modulate heparanase expression. PMA and ionomycin, potent inducers of leukocyte activation, can stimulate heparanase expression in lymphocytes [61], neutrophils, and platelets [50,124]. The viral RNA mimetic poly(I:C) can also upregulate heparanase in natural killer cells [50]. By upregulating heparanase during immune cell activation, these compounds enable heparanase-facilitated leukocyte functions such as cytokine production [152,153] and migration [50,51,153]. These findings add to the growing body of literature on the importance of heparanase in immune cell function, however, more work is needed to fully define its importance in immunity.

Radiation has also been shown to increase heparanase expression. UVB irradiation of human skin samples and cultured keratinocytes induced heparanase expression and activity [125] and rats with liver cirrhosis that received partial liver irradiation showed an upregulation of the heparanase proenzyme in liver and serum [126]. These findings suggest heparanase may be a useful biomarker when monitoring response to radiation. Furthermore, as with chemoresistance, and the recently identified survival signature associated with heparanase [154], heparanase upregulation may be another example of heparanase-mediated therapeutic resistance. The upregulation of heparanase upon treatment with these therapeutics may mean that combining with heparanase inhibitors could have synergistic benefits for anti-cancer treatments.

## 3. Regulation of Heparanase Enzymatic Activity: Proteolytic Activation and Natural Inhibitors

Heparanase is synthesized as an inactive proenzyme containing an 8 kDa and a 50 kDa subunit sequence joined by a linker sequence. This proenzyme then undergoes proteolytic processing by cathepsin L to remove the linker sequence and allow the heterodimerization of the two subunits to become an active enzyme [155,156]. Cathepsin L expression and consequent heparanase activation have been linked to viral infection [118,122] and pancreatitis [127]. Interestingly, in a model of acute pancreatitis, cathepsin L has also been shown to be regulated by heparanase, representing a self-sustaining loop which generates continuous heparanase activity [127]. In addition to cathepsin L, other proteases such as cysteine proteases, cathepsin B, D, S, and other aspartic proteases may also contribute to the activation of heparanase [155]. The existence of this proenzyme containing the linker sequence represents an efficient mechanism for rapid heparanase activation upon certain stimuli.

Heparanase enzymatic activity is also regulated by naturally occurring heparanase inhibitors. Although eosinophils produce heparanase, heparanase enzymatic activity in both resting and activated eosinophils is not detected. This is because eosinophils also express major basic protein which completely inhibits heparanase activity [157]. Two other eosinophil proteins, peroxidase and eosinophil cationic protein, also partially inhibit heparanase activity [157]. HS-interacting protein is also recognized as a natural endogenous heparanase inhibitor [158,159]. HS-interacting protein binds HS on the cell surface and ECM, thus blocking heparanase access. Heparanase-2, the inactive homolog to the active enzyme, can also bind HS, in fact, with higher affinity than the enzymatically active heparanase to indirectly inhibit activity. Heparanase-2 has also been shown to directly interact with heparanase, and thus inhibit heparanase activity directly [160]. Heparin is another well-described natural inhibitor of heparanase activity. Solely expressed by mast cells, this highly sulfated form of HS inhibits heparanase activity by binding directly to the enzyme’s active site [161,162,163]. Finally, heparanase enzymatic activity is affected by pH; enzymatic activity is limited to an acidic microenvironment, e.g., at sites of inflammation or in the core of solid tumors. The optimal pH for heparanase activity is 5.5 and no enzymatic activity is detected at a pH below 3.5 or above 7.0 [164,165,166].

## 4. Heparanase in Regulating Gene Expression, Protein Expression, and Protein Phosphorylation

### 4.1. Nuclear Heparanase Regulates Gene Transcription

**Figure 2:**
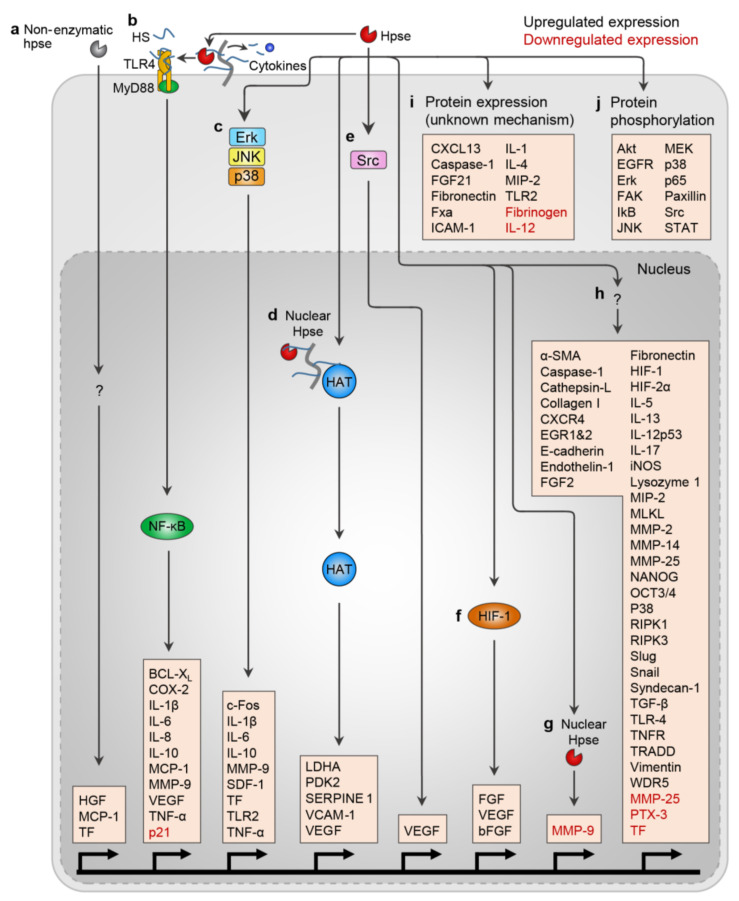
Heparanase regulates protein expression and phosphorylation via multiple mechanisms. Heparanase regulates gene transcription via (**a**) non-enzymatic mechanisms, (**b**) generating soluble HS fragments which bind and activate TLR4 signaling and NF-κB activation, (**c**) Erk, p38, and JNK signaling, (**d**) nuclear localization and cleavage of HS to activate histone acetyltransferase (HAT), (**e**) Src, (**f**) hypoxia inducible factor (HIF)-1 activation, (**g**) direct binding to promoter to block gene transcription, and (**h**) by unknown mechanisms. (**i**) Heparanase can also regulate the expression of other proteins, although the mechanism of this is unknown. (**j**) Finally, heparanase can induce phosphorylation of several proteins. Hpse, heparanase; HS, heparan sulfate.

In addition to its many well-recognized functions, heparanase can also regulate gene expression via multiple direct and indirect mechanisms (Figure 2). Heparanase can enter the nucleus to modify nuclear HS and even exert direct effects on gene transcription. Indeed, heparanase has been shown to enter the nucleus of myeloma cells and cleave nuclear HS on syndecan-1 [167]. Nuclear HS inhibits histone acetyltransferases (HATs), thereby inhibiting gene transcription [168]. By entering the nucleus and degrading nuclear syndecan-1, heparanase mediates HAT activation and transcription of genes associated with an aggressive tumor phenotype [168]. Conversely, nuclear heparanase has also been shown to bind non-specifically to DNA and compete for binding with NF-κB, thus preventing transcription of many NF-κB target genes and acting as a tumor suppressor [169]. Heparanase has also been identified in the nucleus of human glioma and breast cancer cell lines and in patient samples of squamous cell carcinoma [170] and adenocarcinoma [171]. Chromatin immunoprecipitation experiments revealed that heparanase is recruited to promoters and 5′ coding regions of microRNA genes miR-9 and miR-183 (previously implicated in cancer and epithelial-mesenchymal transition (EMT)) and other genes linked to development and differentiation pathways [172]. These studies suggest that in neoplastic cells, nuclear heparanase acts to drive tumor aggressiveness and heparanase localization in the nucleus can correlate with poor patient prognosis [171,173,174].

Furthermore, in human Jurkat T cells, heparanase controls nuclear histone H3 methylation patterns to regulate expression of the immune response genes CD69, IL-2, and IFN-γ [172]. Heparanase also contains two potential nuclear localization sequences, and enzymatically active heparanase has been found in the chromatin compartment of the nucleus, where it co-localizes with RNA polymerase II in T cells [172]. This nuclear heparanase positively controls the transcription of several genes in T cells important for immune function.

### 4.2. Heparanase Regulates Gene and Protein Expression and Protein Activation

The expression of heparanase is tightly regulated by many factors as described above. In contrast, heparanase itself is also involved in the regulation of different genes that contribute to a variety of physiological processes as well as disease settings. It has been reported that the expression of growth factors such as VEGF, HGF, bFGF, FGF-2, and transforming growth factor-β/β1 which play essential roles in EMT, bone formation, angiogenesis, tumor angiogenesis, and renal diseases, are regulated by heparanase. This effect of heparanase is observed in both in vivo and in vitro studies and is through either its enzymatic or non-enzymatic activities [76,168,175,176,177,178,179,180,181].

Heparanase can also alter the expression of EMT gene markers such as Slug, Snail, vimentin, α-SMA, Fibronectin, Collagen-1, Cathepsin-L, Endothelin-1, and E-cadherin as well as stem cell markers (CXCR4, OCT3/4, and NANOG) which further contribute to the pathological processes such as acute kidney disease and gastric adenocarcinoma [179,181,182]. In addition, considerable evidence supports a role for heparanase in regulating genes encoding pro-inflammatory cytokines, chemokines, and other proteins involving macrophage activation, function, and polarization, namely IL-1b, IL-6, IL-10, IL-12p53, TNF-α, MIP-2, toll-like receptor-2 (TLR-2), TLR-4, iNOS, c-Fos, CXCL-12, lysozyme 1, VEGF-A, and caspase-1. The expression of these molecules as well as the activation of macrophages play important roles in diseases such as colitis-associated tumorigenesis [131], ulcerative colitis [131], and acute kidney injury [182].

It is well-documented that heparanase overexpression occurs in most malignancies and is involved in tumor progression and prognosis. Here, heparanase contributes to the regulation of tumor-related processes, such as angiogenesis, inflammation, and tumor cell invasion and metastasis, reviewed in detail recently [64]. Heparanase has the ability to modify the expression of genes involved in these tumor-related processes including IL-17A [84], MCP-1 [183], MMPs [76,79,168,169,184], TNF-α [153,169], VEGF [76,168,175,177], and VEGF-C [185]. It is worth noting that heparanase also plays an important role in regulating the expression of many different inflammation-related genes such as IL-1β, IL-5, IL-6, IL-8, IL-10, IL-13, and vascular cell adhesion molecule 1 (VCAM-1) [51,120,152,183,186]. Moreover, the silencing or overexpression of heparanase also impacts the expression of other ECM-degrading enzyme MMPs such as MMP-2, MMP-9, MMP-14, and MMP-25, which affect migration of immune cells to inflammatory sites. Heparanase-induced upregulation or downregulation of these genes seems to vary depending on the disease [51,76,79,168,169,184]. The involvement of heparanase in the regulation of genes contributing to different physiological and pathological processes is listed in Table 3.

A recent study has also used transcriptomics to show that heparanase negatively regulates a number of genes involved in defense responses to viruses [201]. Following infection with HSV-1, differences in the transcriptomic landscape of wild-type and heparanase knock-out cells were observed. Heparanase knock-out cells were enriched in genes related to an antiviral and innate immune response (such as Interferon regulatory factors), while infected wild-type cells were enriched for genes involved in gene expression and processing, and hence viral replication. This suggests heparanase dampens the host’s antiviral defense response while simultaneously enhancing the virulence of HSV-1. As described above, heparanase is upregulated during infection with several types of viruses. Thus, heparanase upregulation and downstream gene regulation are likely a mechanism of viral pathogenicity. Genes involved in response to viral infection were not the only genes found to be modulated by heparanase in this study. Heparanase was also found to positively regulate genes involved in blood vessel development, cell-cell adhesion, inflammatory response, ECM reorganization, and leukocyte chemotaxis, and negatively regulate genes in pathways related to an antiviral defense response, regulation of viral genome replication, antigen processing and presentation, regulation of nuclease activity, and activation of an immune response [201]. Similarly, transcriptomic analysis has also been performed on heparanase-silenced melanoma cells [154]. This study found heparanase to negatively regulate genes relating to many pathways, including signaling, communication, response to cytokines, protein phosphorylation, cell adhesion, inflammatory response, and apoptotic processes. These two studies highlight the broad regulatory role heparanase plays in several cellular pathways.

As mentioned above, heparanase can directly and indirectly alter the expression of numerous genes. Since gene expression does not always correlate with protein expression, validating that expression changes occur at the protein level is important, and for heparanase-regulated genes, this is often the case. In addition to regulating EMT-related genes at the transcriptional level as mentioned above, heparanase also contributes to the expression of these genes at the protein level. This was demonstrated by the increased expression of α-SMA, fibronectin, and vimentin in transgenic mice over-expressing heparanase at both the mRNA and protein level [179]. There are many other examples of specific protein expression shown to be regulated by heparanase at the transcriptional level. Depletion of heparanase or employing heparanase inhibitors in either mouse models or cell lines resulted in the reduced expression of growth factors, cytokines, and other proteins such as bFGF, VEGF, HGF [76,176,177], CXCL2, TLR2 [153], and IL-17A [84]. These proteins play a key role in the progression of different tumor types. In the presence of heparanase, pro-inflammatory cytokines IL-6, IL-10, MCP-1, and TNF-α are elevated at the mRNA and protein level in both human and mouse immune cells in vivo. These cytokines are implicated in autoimmune diseases such as atherosclerosis and autoimmune encephalitis [199,200]. Heparanase, by modifying the levels of these cytokines, is therefore also involved in mediating these diseases. We have summarised the list of proteins and the processes and diseases involved that are influenced by heparanase in Table 3.

Other reports have also shown that the levels of specific proteins are altered as a result of heparanase expression, e.g., IL-4 [51,200], CXCL1 [183], and fibrinogen [198], however, mRNA expression levels have not been determined for these proteins. Whilst it is still unknown how heparanase regulates the expression of these proteins, based on the other examples listed above, it can be predicted that heparanase modulates expression of these proteins by altering the expression and secretion of signaling molecules (e.g., cytokines) that ultimately alter gene expression and consequently protein levels.

### 4.3. Protein Phosphorylation

Protein phosphorylation is an important biological process whereby many receptors and enzymes are activated or deactivated by phosphorylation or dephosphorylation, respectively. Several studies have demonstrated that heparanase can indirectly regulate protein phosphorylation (Figure 2). Akt, a member of AGC kinases, is associated with cellular signaling pathways related to cell proliferation, cell growth, cell survival, and metabolism [202]. Heparanase has been suggested to induce Akt phosphorylation in endothelial cells, macrophages, fibroblasts, and various tumor-derived cells [76,199,203,204,205]. It seems that Akt phosphorylation requires enzymatic activity of heparanase since blocking heparanase activity reduced levels of Akt phosphorylation [76,79]. Heparanase is also involved in the phosphorylation of ERK, another kinase involved in numerous cellular functions such as proliferation, survival, apoptosis, motility, transcription, metabolism, and differentiation [206]. Again, heparanase has been shown to enhance ERK phosphorylation levels in macrophages and myeloma cell lines through its enzymatic activity [76,153]. Increased ERK phosphorylation is also observed in neural stem and progenitor cells overexpressing heparanase during cell differentiation [207]. Heparanase is also implicated in mediating EGFR phosphorylation, where EGFR signaling is a key regulator of cell growth, cell migration, proliferation, and cell survival [208,209]. It is reported that overexpression of heparanase also stimulates the phosphorylation of EGFR in different tumor cell lines [205,209] and inhibiting heparanase expression results in the reduction of EGFR phosphorylation [79].

In addition to Akt, ERK, and EGFR, heparanase is suggested to mediate the phosphorylation of Signal Transducer and Activator Of Transcription (STAT) proteins including STAT3 and STAT5b. In a tumor setting, heparanase enhances the phosphorylation of STAT3 and STAT5b. Notably, increased cytoplasmic pSTAT3 is associated with larger tumor size and reduced patient survival in a cohort of patients with head and neck squamous cell carcinoma [205]. The increased STAT3 phosphorylation is eliminated in pancreatic cells isolated from mice treated with a heparanase inhibitor, which further strengthens the involvement of heparanase in STAT3 phosphorylation. Additionally, heparanase regulates the levels of phosphorylated Focal-adhesion kinase (FAK), SRC, and paxillin, adhesion molecules required for tumor cell cluster formation, the process that facilitates cancer metastasis [197]. In a mouse model of acute pancreatitis, heparanase overexpression resulted in elevated levels of IκB phosphorylation and correlated with increased TNF-α expression. A similar observation was noted for IL-6 and STAT3 phosphorylation which indicates the association of heparanase with the activation of key signaling pathways related to acute pancreatitis [127]. Furthermore, heparanase can also stimulate the phosphorylation of p65 NF-κB [131], p38, and JNK, which lead to the activation of NF-κB and the induction of cytokine expression in macrophages [210]. Proteins of which their phosphorylation state is regulated by heparanase are listed in Table 4.

## 5. Conclusions

Heparanase is widely considered a key player in several diseases including cancer, heart disease, and viral infection. Thus, the clinical inhibition of heparanase provides a potential method to treat these diseases. Understanding its intricate role in these diseases is key to designing effective treatments. This review highlighted the many molecular regulators of heparanase in different disease contexts. The array of different molecules, pathways and settings that regulate heparanase expression illustrate the diversity of heparanase expression and functions during disease. We also discuss how heparanase itself can regulate the expression of many downstream genes as well as the phosphorylation of proteins, and thus regulate the activity of several pathways, making heparanase a master regulator of several cellular processes in physiology and disease. Furthering our understanding of how heparanase itself is regulated, as well as the greater heparanase regulatory network, will help to develop treatments for heparanase-mediated diseases.

## Figures and Tables

**Table 1:** Mammalian heparan sulfate-binding proteins.

Protein	Technique	Reference
Amyloid peptide β (1–42)	Surface plasmon resonance	[13]
Amyloid precursor protein	Fluorescence lifetime imaging microscopy	[14]
Annexin V	Affinity chromatography	[15]
Basic fibroblast growth factor (bFGF) (FGF-2)	Iodinated-bFGF and specific activity following heparanase addition; Affinity chromatography; Iodinated-bFGF and specific activity; Cross-linking of iodinated-bFGF following heparitinase treatment	[16,17,18,19]
Collagen I	Affinity chromatography; Surface plasmon resonance	[13,20]
Collagen V	Solid phase binding assay; Surface plasmon resonance	[21,22]
Collagenase IV	Antibody-linked detection assay; Surface plasmon resonance	[13,23]
Collagen VI	Surface plasmon resonance	[13]
chemokine (C-X-C motif) ligand (CXCL1) (KC)	Surface plasmon resonance	[24]
CXCL2 (MIP-2)	Surface plasmon resonance	[24]
CXCL6 (GCP-2)	Surface plasmon resonance	[24]
CXCL10 (IP-10)	Alkaline phosphatase-conjucated IP-10; Surface plasmon resonance	[24,25]
CXCL11 (I-TAC)	Surface plasmon resonance	[24]
CXCL13	Surface plasmon resonance	[26]
Endostatin	Alkaline phosphatase-endostatin binding assay; Filter-binding assay; Surface plasmon resonance	[13,27,28]
FGFR4	Affinity chromatography	[29]
Fibronectin	Affinity chromatography; Antibody-linked detection assay	[7,8,23]
HGF	Affinity chromatography	[30]
Histidine-rich glycoprotein	Flow cytometry after heparanase treatment	[31]
High mobility group protein B1	Biotinylation and streptadivin-HRP detection	[32]
Integrin α5β1	Surface plasmon resonance	[13]
Interferon-β (IFN-β)	Filter binding assay	[33]
Interleukin-8 (IL-8)	Affinity co-electrophoresis	[34]
Laminin-1	Antibody-linked detection assay; Surface plasmon resonance	[13,23]
L-selectin	Heparinase treatment of 35SO4-labeled L-selectin ligands and SDS-PAGE; Affinity chromatography	[35,36]
Monocyte chemoattractant protein-1 (MCP-1)	Competitive binding to 3H-heparin by nitrocellulose membrane filtration and liquid scintillation	[37]
Macrophage migration inhibitory factor (MIF)	Flow cytometry of fluorescently labeled MIF on A549 cells after heparinase treatment	[38]
Macrophage inflammatory protein-1α (MIP-1α)	Affinity chromatography after heparinase treatment	[39]
NKp46	ELISA	[40]
Platelet-derived growth factor (PDGF)	Surface plasmon resonance; Affinity chromatography	[41,42]
Platelet Factor 4	Affinity co-electrophoresis	[34]
P-selectin	Affinity chromatography	[36]
Receptor for advanced glycation end products (RAGE)	Biotinylation and streptadivin-HRP detection	[32,43]
Regulated on activation normal T cell expressed and secreted (RANTES) (CCL5) (oligomerized, filamentous)	Surface plasmon resonance	[24,44]
Receptor protein-tyrosine phosphatase-σ (RPTP-σ)	Blot overlay assay probing agrin and collagen with cPTP-σ-conditioned medium following heparinase digestion	[45]
Stromal cell-derived factor-1 (SDF-1)	Flow cytometry of endothelial cells after heparinase treatment for bound SDF-1	[46]
Transglutaminase-2	Surface plasmon resonance	[13]
Thrombospondin-1	Surface plasmon resonance	[13]
Vascular endothelial growth factor (VEGF)	Metabolic labeling of pHEBO cells overexpressing VEGF189 followed by heparinase treatment, immunoprecipitation, and SDS PAGE	[47]

CXCL, C-X-C motif ligand; IP-10, interferon-γ induced protein-10; MCP-1, monocyte chemoattractant protein-1; MIF, Macrophage migration inhibitory factor; MIP-1α, macrophage inflammatory protein-1α; PDGF, platelet-derived growth factor; RAGE, receptor for advanced glycation end products; RANTES, regulated on activation normal T cell expressed and secreted; RPTP-σ, receptor protein-tyrosine phosphatase-σ; SDF-1, stromal cell-derived factor-1.

**Table 2:** Proteins, molecules, pathogens, pathways, and therapeutics that modulate heparanase expression.

Agent	Species	Findings	Reference
Transcription factors			
c-Myc	Human	hTERT enabled binding of c-Myc to the heparanase promoter and increased heparanase mRNA expression	[65]
Early growth response 1 (EGR1)	Human and mouse	Direct binding to the heparanase promoter resulted in activation of the heparanase promoter in PC-3, COLO397, and MCF-7 cells, and repression of the heparanase promoter in MM170 cells; EGR1 was recruited to the heparanase promoter upon glucose treatment of HEK293T cells	[61,62,66,67,68]
Erythroblast Transformation Specific 1 (ETS1) and ETS2	Human	Direct binding to the heparanase promoter increased heparanase mRNA expression	[69]
GA-binding protein (GABP)	Human	Direct binding to the heparanase promoter increased heparanase promoter activity	[70,71]
NF-κB	Human and mouse	NF-κB-deficient lung carcinoma cells produced less heparanase; Inhibiting canonical NF-κB signaling blocked tumor necrosis factor-α (TNF-α)-induced upregulation of heparanase in endothelial cells; Chemotherapy treatment of multiple myeloma cells, hypoxia induction in pancreatic cancer cells, or infection with herpes simplex virus-1 (HSV-1) activated NF-κB to upregulate heparanase	[72,73,74,75,76]
p53	Human and mouse	Direct binding to the heparanase promoter reduced heparanase mRNA expression	[77]
Snail	Mouse	Overexpression of Snail in B16F1 cells increased heparanase mRNA expression	[78]
specificity protein 1 (SP1) and SP3	Human	Direct binding to the heparanase promoter increased heparanase promoter activity	[70]
MicroRNA			
miR-1258	Human	miRNA-1258 suppressed heparanase expression in breast cancer cells	[79]
miR-1252-5p		Overexpression of miR-1252-5p in multiple myeloma cells reduced heparanase mRNA and protein expression and activity	[80]
Cytokines			
IFN-γ	Human	Treatment of endothelial cells with IFN-γ increased heparanase mRNA expression and activity	[81]
IL-1β	Human and mouse	Treatment of endothelial cells with IL-1β increased heparanase mRNA expression	[73,82]
IL-2	Mouse	Treatment of purified NK cells with IL-2 induced expression of both pro-heparanase and the catalytically active heparanase protein, more so when also cultured with IL-15	[50]
IL-10	Human	IL-10 treatment of SUM149 breast cancer cells modestly increased heparanase mRNA expression	[83]
IL-15	Mouse	Treatment of purified NK cells with IL-15 induced expression of both pro-heparanase and the catalytically active heparanase protein, more so when also cultured with IL-12 and IL-18	[50]
IL-17	Human	Treatment of cervical cancer cells with IL-17 increased heparanase mRNA expression, and IL-17 knockdown reduced heparanase expression	[84]
MCP-1	Mouse	MCP-1 inhibition reduced glomerular heparanase expression	[85]
TNF-α	Human, mouse and bovine	Treatment of endothelial cells, U937 macrophages and colon cancer cells with TNF-α increased heparanase mRNA and protein expression	[73,82,86,87,88]
Growth factors			
bFGF	Human	Treatment of lung cancer cells with bFGF induced heparanase mRNA expression	[89]
HGF	Human	Treatment of lung cancer cells with HGF induced heparanase mRNA expression; HGF activated the PI3K/Akt/NF-κB signaling pathway and upregulated heparanase mRNA and protein expression	[89,90]
FGF23	Human	Treatment of multiple myeloma cells with FGF23 increased heparanase mRNA expression, likely via Egr1 upregulation which was also upregulated	[91]
PDGF	Human	Treatment of lung cancer cells with PDGF induced heparanase mRNA expression	[89]
VEGF	Human	Treatment of endothelial cells with VEGF reduced heparanase expression; Treatment of melanoma cells with recombinant VEGF increased heparanase mRNA expression, and VEGF knockdown decreased heparanase expression	[82,92]
Pathways			
CD3/CD28 activation	Human and mouse	Heparanase mRNA and protein increased with anti-CD3 and anti-CD28 antibody stimulation in mouse splenic (CD4/CD8) and human PBMC-derived T cells	[62,93]
MEK/ERK pathway	Human	Activation of the MEK/ERK pathway increased heparanase expression	[66]
Wnt signaling	Hamster and Zebrafish	CHO-K1 cells treated with lithium chloride (LiCl; a Wnt signaling activator) modestly increased heparanase protein expression. Zebrafish embryos treated with LiCl increased heparanase mRNA expression	[94]
Other biological molecules			
Heparin	Hamster	CHO-K1 cells treated with heparin increased heparanase mRNA, protein, and enzymatic activity. Authors propose this may be via Wnt signaling	[94]
Lumican	Mouse	Treatment of B16F1 cells with recombinant lumican increased heparanase mRNA and protein expression; Treatment of Snail-overexpressing B16F1 cells (which resulted in increased heparanase expression) with recombinant lumican decreased heparanase mRNA expression but did not change protein expression	[78]
Hormones, metabolites and other signaling molecules			
Advanced Glycation End Products (AGEs)	Human	Exposure of HMVECs to AGEs increased heparanase mRNA and protein expression	[95]
Basic protein	Rat	T lymphocytes degraded heparan sulfate (HS) after stimulation with Con A or basic protein	[96,97,98]
BRAF	Human	Wild tpye BRAF suppressed ETS1 family of transcription factors, which suppressed heparanase promoter activity and mRNA expression. Mutant BRAF lost repression ability and heparanase mRNA expression was upregulated	[71]
Endothelial nitric oxide synthase (eNOS)	Mouse	In a rat model of adriamycin nephropathy, the deletion or inhibition of eNOS induced heparanase mRNA and protein expression	[99]
Estrogen	Human and mouse	Estrogen treatment of ER-positive MCF-7 cells increased heparanase mRNA and protein expression via estrogen receptor signaling and estrogen response elements in the heparanase promoter. Upregulation of heparanase occured more so in low levels than high levels of estrogen; Estrogen supplementation in MCF-7 implanted tumors in mice increased heparanase protein expression; Treatment of cholangiocarcinoma cells with an estrogenic inducer upregulated heparanase mRNA expression; Estrogen treatment of ER-positive EO771 breast cancer cells increased heparanase mRNA expression	[100,101,102,103]
High glucose	Human and bovine	Glucose-treated cells modestly increased heparanase protein expression. Heparanase mRNA expression, secretion, and activity increased upon glucose treatment	[104,105,106]
Hypoxia (1% O2)	Human	Hypoxia-induced activation of NF-κB upregulated heparanase mRNA and protein expression	[74]
Low density lipoprotein (LDL)	Human	Treatment of endothelial cells with LDL increased heparanase mRNA expression	[82]
Lipopolysaccharide (LPS)	Human and mouse	Stimulation of B cells with LPS increased heparanase activity. Activating toll-like receptor 4 (TLR4) with LPS on PBMCs and cord blood cells increased heparanase mRNA expression; LPS stimulation of PBMCs increased heparanase mRNA expression; LPS stimulation of endothelial cells increased levels of enzymatically active heparanase	[63,98,107,108,109]
Oleic acid	Bovine	Treatment of endothelial cells increased heparanase mRNA and protein expression, and was likely via Sp1	[82]
Reactive oxygen species (ROS)	Human and rat	In a rat model of adriamycin nephropathy, the depletion of hydroxyl radicals with DMTU reduced heparanase expression. Inducing mouse podocytes to generate free radicals and ROS increased heparanase mRNA and protein expression. Treatment of endothelial cells with ROS scavengers perturbed glucose-mediated heparanase expression	[106,109,110]
Vitamin D	Rat and mouse	Vitamin D treatment reduced heparanase mRNA expression via initiating direct binding of the vitamin D receptor to the heparanase promoter	[111]
Pathogens			
*Fusobacterium nucleatum*	Human	Co-culture of SSC-25 oral cancer cells with *F. nucleatum* increased heparanase expression	[112]
*Helicobacter pylori*		*H. pylori* infection of gastric cancer cells induced an upregulation of heparanase protein, which was dependent on MAPK signaling	[113]
*Pseudomonas aeruginosa*	Mouse	*P. aeruginosa* intracorneal infection in mice induced an upregulation of heparanase mRNA and enzymatically active protein in the cornea. This was from both infiltrating immune cells as well as from the corneal epithelium	[114]
*Streptococcus pneumoniae*	Mouse	Intranasal *S. pneumoniae* infection in mice increased heparanase protein expression	[115]
Bovine herpes virus	Human	Heparanase mRNA was upregulated upon epithelial cell infection in vitro	[75]
SARS-CoV-2	Human	COVID-19 patients displayed elevated heparanase activity and soluble HS levels in the plasma; Increase shed syndecan-1 was observed	[116,117]
Cytomegalovirus	Human	Heparanase mRNA was upregulated upon fibroblast cell infection in vitro	[75]
Dengue virus	Human	Dengue virus protein NS1 upregulated heparanase protein in endothelial cells, and upregulation was found to be macrophage inhibitory factor-dependent	[118,119]
HSV-1	Human	Heparanase mRNA and protein were upregulated upon HSV-1 infection through NF-κB activation	[75,120]
HSV-2	Human	Heparanase mRNA was upregulated upon epithelial cell infection in vitro	[75]
Porcine reproductive and respiratory syndrome virus	Pig	Piglets infected with PRSSV in vivo increased heparanase mRNA expression in alveolar macrophages Cells infected in vitro with PRSSV increased heparanase mRNA and protein expression	[121,122]
Pseudorabies virus	Human	Heparanase mRNA was upregulated upon epithelial cell infection in vitro	[75]
Therapeutics			
Bortezomib	Human	Treatment of myeloma cells increased heparanase mRNA and protein expression	[76]
Carfilzomib	Human	Treatment of myeloma cells increased heparanase mRNA expression	[76]
Cisplatin	Human	Treatment of mesothelioma cells, gastric cancer cells, and J774 macrophages increased heparanase mRNA expression	[123]
Clarithromycin	Human	Clarithromycin blocked the upregulation of heparanase mRNA induced by bFGF	[89]
Doxorubicin	Human	Treatment of myeloma cells, gastric cancer cells, and J774 macrophages increased heparanase protein expression	[76,123]
Erythromycin	Human	Erythromycin blocked the upregulation of heparanase mRNA induced by PDGF and HGF	[89]
Paclitaxel	Human	Treatment of gastric cancer cells with paclitaxel increased heparanase mRNA expression	[123]
phorbol-12-myristate-13-acetate (PMA)	Human and mouse	Heparanase mRNA expression increased upon stimulation with PMA ionomycin in EL4 T lymphocytes. HS degradation increased after PMA stimulation in neutrophils, human umbilical vein endothelial cells (HUVECs), and platelets; Heparanase mRNA, protein, and activity increased in human NK cells after activation with B-LCL cells, IL-2 and PMA, and ionomycin	[50,61,124]
Poly(I:C)	Mouse	Poly(I:C) stimulation in vivo increased heparanase activity in splenic NK cells	[50]
Radiation	Human and rat	Human epidermal keratinocytes exposed to UVB radiation exhibited increased heparanase enzymatic activity and detectable levels of the 50 kDa active subunit; Rats with experimental liver cirrhosis showed an increase in heparanase precursor protein in liver and serum after treatment with partial liver radiation	[125,126]
Tamoxifen	Human	Treatment of MCF-7 cells with high concentration of tamoxifen inhibited estrogen-induced heparanase expression; Tamoxifen treatment of MCF-7 cells and T47D cells increased heparanase mRNA expression	[100,102]
Miscellaneous			
Cerulein	Mouse	Injection of cerulein into mice increased heparanase mRNA expression and enzymatic activity in pancreatic tissue extracts	[127]

AGE, Advanced glycation end product; DMTU, dimethylthiourea; eNOS, endothelial nitric oxide synthase; ERK, extracellular signal-regulated kinase; ETS, E26 transformation-specific or E-twenty-six; GABP, GA-binding protein; HGF, hepatocyte growth factor; HMVEC, human microvascular endothelial cell; HSV, herpes simplex virus; hTERT, telomerase reverse transcriptase; LDL, low-density lipoprotein; LPS, lipopolysaccharide; MEK, mitogen-activated protein kinase; MCP, monocyte chemoattractant protein; PBMC, peripheral blood mononuclear cell; PI3K, phosphoinositide 3-kinases; SP, specificity protein; TLR4, Toll-like receptor 4; WT, wild type.

**Table 3:** Genes and proteins that are regulated by heparanase.

Gene/Protein	Observation/Mechanism	Related Disease/Function	Reference
Genes			
Aromatase	The expression of aromatase was decreased in heparanase- knockout obese mice. Heparanase was required for the activation of fatty acid-stimulated macrophages to induce aromatase in adipose stromal cells	Obesity-associated breast cancer progression	[103]
Bcl-XL (Bcl2l1)	Increased expression of Bcl-XL in heparanase overexpressing transgenic mice with dextran sulfate sodium (DSS)-induced colitis was regulated by NF-κB	Ulcerative colitis	[131]
Caspase-1	Silence of heparanase and heparanase inhibitor (SST0001) blocked caspase 1 expression in human kidney cells	Acute kidney injury/M1 macrophage polarization	[182]
Cathepsin L	Induction of acute kidney injury in heparanase-transgenic mice enhanced the expression of cathepsin L mRNA. Pre-treatment with heparanase inhibitor PG545 reduced the expression of cathepsin L	Epithelial-mesenchymal transition (EMT)/Acute kidney injury	[179]
CD44	siRNA knockdown of heparanase in SUM149 breast cancer cells reduced mRNA expression of CD44	Breast cancer	[83]
c-Fos (AP-1)	The expression of c-Fos was decreased in heparanase-knockout macrophages and adding exogenous heparanase enhanced c-Fos expression. Heparanase regulated the gene expression of c-Fos through Erk, p38, and JNK signaling pathway	Tumor/Induction of cytokine expression	[153]
Collagen-I	Treatment of KATO-III gastric cancer cells with heparanase inhibitor suramin exhibited reduced expression of collagen-I	EMT/Gastric ring cell adenocarcinoma	[181]
Cox-2	Cox-2 mRNA expression was increased in heparanase overexpressing transgenic mice with DSS-induced colitis and was regulated by NF-κB	Ulcerative colitis	[131]
	Heparanase upregulated the mRNA expression of Cox-2 in cancer cells	Tumor/Promoting angiogenesis	[187]
CXCR-4	mRNA expression of CXCR-4 was decreased in gastric cancer cell KATO-III treated with heparanase inhibitor suramin	EMT/Gastric ring cell adenocarcinoma	[181]
EGR1	Overexpression of heparanase increased Egr1 mRNA expression	Modulation of EGR gene expression	[188]
EGR2	Overexpression of heparanase increased Egr2 mRNA expression	Modulation of EGR gene expression	[188]
E-Cadherin	The expression of Epithelial marker E-cadherin was increased in KATO-III gastric cancer cells treated with heparanase inhibitor suramin	EMT/Gastric ring cell adenocarcinoma	[181]
Endothelin-1 (ET-1)	Induction of acute kidney injury in heparanase-transgenic mice enhanced the expression of ET-1mRNA. Pre-treatment with heparanase inhibitior PG545 reduced the expression of ET-1	EMT/Acute kidney injury	[179]
FGF/bFGF	Heparanase activated HIF1 pathway which led to reduced mRNA expression level of bFGF in heparanase knockdown cells and elevated mRNA expression level of bFGF in heparanase overexpressing-cells	Cervical cancer	[177]
FGF-2	Treatment of human osteoblasts with heparin, a heparanase inhibitor, inhibited mRNA FGF2 expression	Growth of osteoblasts	[180]
Fibronectin (FN)	Heparanase-transgenic mice displayed remarkable upregulation of FN during acute kidney injury. Pre-treatment with heparanase inhibitor PG545 abolished the increased expression of FN in heparanase-transgenic mice	EMT/Acute kidney injury	[179]
	Heparanase-silenced cells showed reduced FN expression; Renal tissue extracts from mice with acute kidney injury treated with Roneparstat showed reduced FN expression	EMT/Acute kidney injury	[182,189]
Hepatocyte growth factor (HGF)	Addition of either recombinant or chemotherapy-generated soluble heparanase increased HGF mRNA expression. Immunodepletion or addition of heparanase inhibitor diminished the increased expression of HGF gene. Upregulation of HGF expression by heparanase was independent of heparanase enzyme activity	Tumor progression	[76,176]
HIF-1	mRNA expression level of HIF1 was reduced in heparanase knockdown cells and increased in heparanase-overexpressing cells	Cervical cancer	[177]
HIF-2α	Knockdown of heparanase in HUVEC cells reduced HIF-2α expression	Tumor angiogenesis	[190]
IL-1β	HS fragments generated by heparanase activated TLR4, MyD88, and NF-κB to upregulate IL-1β mRNA	Inflammation	[120,152]
	The expression of IL-1β in macrophages isolated from heparanase-knockout mice was significantly reduced compared to macrophages isolated from wild type mice. Heparanase regulated IL-1β expression through Erk, p38, and JNK signaling pathway	Tumor/Regulation of cytokine expression in macrophage	[153]
	Increased expression of IL-1β in heparanase overexpressing transgenic mice with colitis-associated carcinoma	Colitis-associated tumor/Induction of NK-κB activation/Macrophage activation	[131]
	Heparanase upregulated the expression of IL-1β in PMA-activated U937 macrophages. Treatment cells with heparanase inhibitor SST0001 reduced IL-1β expression	Acute kidney injury/M1 macrophage polarization	[182]
IL-5	House dust mite (HDM)-induced allergic inflammation in heparanase deficient mice reduced mRNA expression of IL-5 in lung cells	Allergic inflammation/Recruitment of eosinophils and mucus-secreting airway epithelial cells	[51]
IL-6	HS fragments generated by heparanase activated TLR4, MyD88, and NF-κB to upregulate IL-6	Inflammation	[152]
	The expression of IL-6 in macrophages isolated from heparanase deficient mice was significantly reduced compared to macrophages isolated from wild type mice. Heparanase regulated IL-6 expression through Erk, p38, and JNK signaling pathways	Tumor/Regulation of cytokine expression in macrophage	[153]
	IL-6 mRNA expression was increased in heparanase transgenic mice with DSS-inducedcolitis. LPS-treated mouse peritoneal macrophages increased mRNA expression of IL-6 in the presence of recombinant enzymatically active heparanase	Ulcerative colitis/Induction of NK-κB activation/Macrophage recruitment and activation	[131]
	Induction of acute kidney injury in heparanase-transgenic mice enhanced the expression of mRNA IL-6. Pre-treatment with heparanase inhibitior PG545 reduced the expression of IL-6	EMT/Acute kidney injury	[179]
	Heparanase upregulated the expression of IL-6 in PMA-activated U937 macrophage cells. Treatment of cells with heparanase inhibitor SST0001 reduced IL-6 expression	Acute kidney injury/M1 macrophage polarization	[182]
	Heparanase induced the expression of IL-6 by fatty acid-stimulated macrophages in a dose-dependent manner	Obesity-associated breast cancer	[103]
	IL-6 expression was increased in heparanase-knockout macrophages treated with exogenous heparanase and chemotherapy	Tumor Growth/Induction of pro-inflammatory cytokine expression by chemotherapy-treated macrophage	[123]
IL-8	HS fragments generated by heparanase activated TLR4, MyD88, and NF-κB to upregulate IL-8	Inflammation	[152,183]
IL-10	IL-10 mRNA expression was reduced in chemotherapy-treated macrophages isolated from heparanase knockout mice	Tumor Growth/Induction of pro-inflammatory cytokine expression by chemotherapy-treated macrophage	[123]
	HS fragments generated by heparanase activated TLR4, MyD88, and NF-κB to upregulate IL-10	Inflammation	[152]
	The expression of IL-10 in macrophages isolated from heparanase deficient mice was significantly reduced compared to macrophages isolated from wild type mice. Heparanase regulated IL-10 expression through Erk, p38, and JNK signaling pathway	Tumor/Regulation of cytokine expression in macrophage	[153]
	Inhibition of heparanase with SST0001 reduced IL-10 mRNA expression in macrophages	Acute kidney injury/M1 macrophage polarization	[182]
IL-13	(HDM-induced allergic inflammation in heparanase deficient mice reduced mRNA expression of IL-13 in lung cells	Allergic inflammation/Recruitment of eosinophils and mucus-secreting airway epithelial cells	[51]
IL-12p53	LPS-treated mouse peritoneal macrophages increased mRNA expression of IL-12p53 in the presence of recombinant enzymatically active heparanase	Ulcerative colitis/Macrophage activation	[131]
IL-17A	Silencing of heparanase resulted in a significant decrease in the mRNA expression of IL-17A in human cervical cancer cell lines HeLa and SiHa	Promoting tumor angiogenesis, cell proliferation, and invasion in cervical cancer	[84]
Inducible nitric oxide synthase (iNOS)	Heparanase upregulated the expression of iNOS in PMA-activated U937 cells. Treatment of cells with heparanase inhibitor SST0001 suppressed iNOS expression	Acute kidney injury/M1 macrophage polarization	[182]
Lysozyme 1	Heparanase-knockout mice showed less lysozyme 1 expression	Tumor/Macrophage cytotoxic activity is decreased in the absence of heparanase	[153]
MCP-1/CCL-2	Non-enzymatic heparanase in colorectal cancer cell lines could upregulate the expression of MCP-1	Promoting extravasation of colon carcinoma cells	[183]
MIP-2 (CXCL2)	Macrophages isolated from heparanase-deficient mice and mice treated with heparanase-neutralizing antibodies exhibited reduced MIP-2 expression	Tumor/Regulation of cytokine expression in macrophage	[153]
	MIP-2 mRNA expression was reduced in chemotherapy-treated macrophages isolated from heparanase knockout mice	Tumor Growth/Induction of pro-inflammatory cytokine expression by chemotherapy-treated macrophage	[123]
Mixed Lineage Kinase Domain Like Pseudokinase (MLKL)	Transwell co-culture of heparanase-silenced hepatocellular carcinoma (HCC) cells with HUVECs protected HUVECs from MLKL mRNA and protein upregulation and necroptosis In transwell co-cultures of heparanase-overexpressing HCC cells and HUVECs, HUVECs displayed higher MLKL protein expression after co-culture compared to controls	Necroptosis	[191]
matrix metalloprotease-2 (MMP-2)	The mRNA expression of MMP-2 was decreased in the kidney of heparanase deficient mice	Allergen-induced inflammation/DC migration	[51]
	Human melanoma cells deficient in heparanase exhibited increased MMP-2 expression	Melanoma progression	[169]
	Inhibiting heparanase with either PG545 or PI-88 in patient-derived explants of normal mammary tissue increased MMP-2 mRNA expression	Tissue density and breast cancer	[192]
	siRNA knockdown of heparanase in SUM149 breast cancer cells reduced MMP-2 mRNA expression	Breast cancer	[83]
MMP-9	Addition of recombinant or chemotherapy-generated soluble heparanase elevated the expression of MMP-9 in myeloma cells. Chemotherapeutic induction of MMP-9 required heparanase through Erk phosphorylation	Tumor progression	[76,79,184]
	The gene expression level of MMP-9 in heparanase-silenced human kidney 2 (HK2) cells was lower than wild type cells	Renal fibrosis	[193]
	Heparanase upregulated the expression of MMP-9 by its HS-degrading activity and stimulating HAT activity	Myeloma tumor/Upregulation of HAT activity	[168]
	Human melanoma cells deficient in heparanase exhibited increased MMP-9 expression	Melanoma progression	[169]
MMP-14	The mRNA expression of MMP-14 was decreased in the liver of heparanase deficient mice	Allergen-induced inflammation/DC migration	[51]
	Inhibiting heparanase with either PG545 or PI-88 in patient-derived explants of normal mammary tissue increased MMP-14 mRNA expression	Tissue density and breast cancer	[192]
MMP-25	The mRNA expression of MMP25 was increased in the spleen but decreased in mouse bone marrow-derived DCs and Langerhans cells from heparanase deficient mice	Allergen-induced inflammation/DC migration	[51]
NANOG	mRNA expression of NANOG was decreased in KATO-III gastric cancer cells treated with heparanase inhibitor suramin	EMT/Gastric ring cell adenocarcinoma	[181]
OCT3/4	mRNA expression of OCT3/4 was decreased in KATO-III gastric cancer cells treated with heparanase inhibitor suramin	EMT/Gastric ring cell adenocarcinoma	[181]
P21	Heparanase downregulated p21 in colon carcinoma cells through its enzymatic activity and involved TLRs and NF-κB signaling	Colon carcinoma/Modification of cell cycle	[194]
P38	In transwell co-cultures of heparanase-silenced HCC cells and HUVECs, HUVECs displayed lower p38 mRNA and phosphorylated protein expression after co-culture compared to controls	Necroptosis	[191]
PDK2	Nuclear heparanase regulated the mRNA expression of PDK2 through HAT activation. Depletion of heparanase reduced the expression of PDK2 mRNA	Glucose metabolism	[186]
Pentraxin 3 (PTX-3)	Human melanoma cells deficient in heparanase exhibited increased PTX-3 expression	Melanoma progression	[169]
Receptor interacting protein kinase 1 (RIPK1) and RIPK3	Transwell co-cultures of heparanase-silenced HCC cells with HUVECs protected HUVECs from RIPK1 and RIPK3 mRNA and protein upregulation and necroptosis. In transwell co-cultures of heparanase-overexpressing HCC cells and HUVECs, HUVECs displayed higher RIPK1 and RIPK3 protein expression after co-culture compared to controls	Necroptosis	[191]
SDF-1 (CXCL-12)	SDF-1 expression was reduced in heparanase-deficient macrophages. Heparanase regulated SDF-1 expression through Erk, p38, and JNK signaling pathway	Tumor/Promoting phagocytic capacity of macrophages	[153]
SERPINE1	Heparanase regulated HAT activity, leading to upregulation of SERPINE1	Inflammation	[186]
Slug	KATO-III gastric cancer cells treated with heparanase inhibitor suramin reduced Slug mRNA expression	EMT/Gastric ring cell adenocarcinoma	[181]
α-SMA	Treatment of KATO-III gastric cancer cells with heparanase inhibitor suramin exhibited reduced expression of α-SMA	EMT/Gastric ring cell adenocarcinoma	[181]
	Heparanase-overexpressing micedisplayed remarkable upregulation of α-SMA during acute kidney injury. Pre-treatment with heparanase inhibitor PG545 abolished the increased expression of α-SMA in hpse-tg mice	EMT/Acute kidney injury	[179]
	Heparanase-silenced cells showed reduced α-SMA expression	EMT/Acute kidney injury	[182,189]
Snail	Heparanase-silenced cells showed reduced Snail expression	EMT/Acute kidney injury	[182]
Syndecan-1	Inhibiting heparanase with either PG545 or PI-88 in patient-derived explants of normal mammary tissue reduced syndecan-1 mRNA expression	Tissue density and breast cancer	[192]
	In transwell co-cultures of heparanase-silenced HCC cells and HUVECs, HUVECs displayed lower syndecan-1 mRNA and protein expression after co-culture compared to controls. In transwell co-cultures of heparanase-overexpressing HCC cells and HUVECs, HUVECs displayed higher syndecan-1 mRNA and protein expression after co-culture compared to controls	Necroptosis	[191]
Tissue factor (TF)	mRNA expression levels of TF were elevated in heparanase transfected breast carcinoma cells and transgenic mice over-expressing heparanase. Exogenous addition of heparanase also induced TF expression in human promyelocytic leukemia cells. Heparanase induced TF expression via inducing p38 signaling non-enzymatically	Blood coagulation	[195]
	Human melanoma cells deficient in heparanase exhibited increased TF expression	Melanoma progression	[169]
Transforming growth factor (TGF)-β/TGFβ1	Gene expression levels of TGF-β was decreased in the heparanase-silenced tubular cells	EMT/Renal fibrosis	[178,189]
	Induction of acute kidney injury in heparanase-transgenic mice enhanced the expression of TGF-β mRNA. Pre-treatment with heparanase inhibitior PG545 abolished the elevation in TGF-β	EMT/Acute kidney injury	[179]
	Heparanase inhibitor suramin down-regulated TGFβ-1 expression in KATO-III gastric cancer cells	EMT/Gastric ring cell adenocarcinoma	[181]
TLR-2	The expression of TLR-2 in macrophages isolated from heparanase deficient mice and in macrophages isolated from mice treated with heparanase-neutralizing antibodies was significantly reduced. Heparanase regulated TLR2 expression through Erk, p38, and JNK signaling pathway	Tumor/Macrophage activation and function in tumorigenesis	[153]
TLR-4	The expression of TLR-4 on macrophages was upregulated in the presence of heparanase but was reduced when cells were treated with heparanase inhibitor SST0001	Acute kidney injury/Regulation of macrophage polarization	[182]
TNF-α	TNF-α expression was reduced in macrophages isolated from heparanase-knockout mice and in macrophages isolated from mice treated with heparanase-neutralizing antibodies. Heparanase regulated TNF-α expression through Erk, p38, and JNK signaling pathway	Tumor/Macrophage activation and function in tumorigenesis	[153]
	Heparanase overexpressing transgenic mice expressed more TNF-α during DSS-induced colitis through NF-κB signaling. LPS-treated mouse peritoneal macrophages increased mRNA expression of TNF-α in the presence of recombinant enzymatically active heparanase	Ulcerative colitis/Induction of NK-κB activation/Macrophage recruitment and activation	[131]
	Induction of acute kidney injury in heparanase-transgenic mice enhanced the expression of TNF-α mRNA. Pre-treatment with heparanase inhibitior PG545 reduced the expression of TNF-α	EMT/Acute kidney injury	[179]
	Human melanoma cells deficient in heparanase exhibited increased TNF-α expression	Melanoma progression	[169]
	Heparanase upregulated the expression of TNF-α in PMA-activated U937 macrophage cells. Treatment of cells with heparanase inhibitor SST0001 reduced TNF-α expression	Acute kidney injury/M1 macrophage polarization	[182]
	HS fragments generated by heparanase activated TLR4, MyD88, and NF-κB to upregulate TNF-α	Inflammation	[152]
	TNF-α mRNA expression was reduced in chemotherapy-treated macrophages isolated from heparanase knockout mice In transwell co-cultures of heparanase-silenced HCC cells and HUVECs, HUVECs displayed lower TNF-α mRNA and protein expression compared to controls	Tumor Growth/Induction of pro-inflammatory cytokine expression by chemotherapy-treated macrophage	[123]
	In transwell co-cultures of heparanase-overexpressing HCC cells and HUVECs, HUVECs displayed higher TNF-α mRNA and protein expression after co-culture compared to controls	Necroptosis	[191]
TNF-α receptor (TNFR)	In transwell co-cultures of heparanase-silenced HCC cells and HUVECs, HUVECs displayed lower TNFR mRNA and protein expression compared to controls	Necroptosis	[191]
TNFR-associated death domain protein (TRADD)	In transwell co-cultures of heparanase-silenced HCC cells and HUVECs, HUVECs displayed lower TRADD mRNA and protein expression after co-culture compared to controls	Necroptosis	[191]
Vascular cell adhesion molecule 1 (VCAM-1)	Heparanase regulated HAT activity, leading to upregulation of VCAM-1	Inflammation	[186]
VEGF	Heparanase overexpression or exogenous addition led to the enhanced expression of VEGF. Heparanase regulated the expression of VEGF by mediating the activation of SRC family members	Promoting angiogenesis in tumor	[175]
	Heparanase upregulated the expression of VEGF through its HS-degrading activity and stimulating the HAT activity	Tumor phenotype/Upregulation of HAT activity	[168]
	Heparanase regulated the expression of VEGF via activating HIF1 pathway	Cervical cancer	[177]
	Addition of recombinant or chemotherapy-generated soluble heparanase elevated the expression of VEGF in myeloma	Tumor progression	[76]
	Heparanase overexpression in melanoma cell lines increased the expression of VEGF mRNA. Downregulation of heparanase via anti-heparanase siRNA transfection resulted in a significant reduction of VEGF mRNA expression in melanoma cell lines	Melanoma progression	[92]
VEGF-A	Reduced VEGF-A expression was observed in macrophages isolated from heparanase-knockout mice and in macrophages isolated from mice treated with heparanase-neutralizing antibodies	Tumor/Macrophage activation and function in tumorigenesis	[153]
	Heparanase regulated HAT activity, leading to upregulation of VEGF-A	Atherosclerosis/Glucose Metabolism	[186]
VEGF-C	Overexpression of heparanase increased VEGF-C mRNA expression	Pancreatic cancer/Facilitating cell invasion	[185]
Vimentin	KATO-III gastric cancer cells exhibited reduced Vimentin expression after treating with heparanase inhibitor suramin	EMT/Gastric ring cell adenocarcinoma	[181]
	Heparanase-overexpressing mice displayed remarkable upregulation of vimentin during acute kidney injury. Pre-treatment with heparanase inhibition abolished the increased expression of vimentin in heparanase-overexpressing mice.	EMT/Acute kidney injury	[179]
	Heparanase-silenced cells reduced vimentin expression	EMT/Acute kidney injury	[189]
WDR5	Upon paclitaxel treatment, WDR5 expression was induced in wild type but not heparanase-knockout macrophages, but could be rescued with exogenous heparanase Heparanase was required for the expression of WDR5 in macrophages	Tumor Growth/Induction of pro-inflammatory cytokine expression by chemotherapy-treated macrophage	[123]
Proteins			
bFGF	bFGF protein expression was decreased in heparanase knockdown cell and increased in heparanase overexpressing cells via activating HIF1 pathway	Cervical cancer	[177]
BLC	BLC expression was reduced in macrophages isolated from heparanase-knockout mice	Tumor/Macrophage activation and function in tumorigenesis	[153]
Caspase-1	Heparanase-silenced and heparanase inhibitor SST0001-treated cells reduced caspase-1 expression	Acute kidney injury/M1 macrophage polarization	
Cox-2	Heparanase upregulated the mRNA expression of Cox-2 in cancer cells	Tumor/Promoting angiogenesis	[187]
CXCL1 (KC)	Administration of heparanase increased CXCL1 level in mouse serum	Thoracoabdominal aortic aneurysm/Systemic Inflammation	[196]
	CXCL1 expression was reduced in macrophages isolated from heparanase-knockout mice	Tumor/Macrophage activation and function in tumorigenesis	[153]
	Heparanase-stimulated colon cancer cells released CXCL1	Colon cancer	[183]
FGF21	Heparanase-overexpressing mice had higher FGF21 expression in the blood plasma compared to wild type mice	Diabetes/Glucose homeostasis	[197]
Fibrinogen	High dose heparanase-derived peptides induced a decrease in the level of fibrinogen	Coagulopathy and wound healing/Activation of the coagulation system	[198]
Fibronectin	Protein expression of fibronectin was increased in heparanase-overexpressing mice with acute kidney injury but decreased when pre-treating the mice with heparanase inhibitor PG545	EMT/Acute kidney injury	[179]
FXa	Heparanase-derived peptides enhanced the level of FXa probable through interacting with TF	Coagulopathy and wound healing/Activation of the coagulation system	[198]
Hepatocyte growth factor (HGF)	Addition of soluble heparanase or increased heparanase expression upregulated HGF expression in myeloma cell lines. Knockdown of heparanase reduced HGF expression	Tumor progression	[76,176]
HIF1	HIF1 protein expression was decreased in heparanase knockdown cells and increased in heparanase overexpressing cells via HIF1 pathway	Cervical cancer	[177]
ICAM-1	ICAM-1 expression was significantly increased in heparanase overexpressing human breast cancer cell lines. Likewise, the expression of ICAM-1 was decreased in heparanase-knockout cell lines	Cancer metastasis/Promotion of cell cluster formation by modulating adhesion molecules	[197]
IL-1	Addition or overexpression of heparanase upregulated the expression of IL-1	Atherosclerosis/Macrophage activation	[199]
IL-1β	Administration of heparanase increased IL-1β level in mouse serum	Thoracoabdominal aortic aneurysm/Systemic Inflammation	[196]
	Heparanase upregulated the expression of IL-1β in macrophages. Treatment of cells with heparanase inhibitor SST0001 reduced IL-1β expression	Acute kidney injury/M1 macrophage polarization	[182]
	Heparanase via its enzymatic activity upregulated IL-1β through TLR4 signaling	Inflammation	[152]
IL-4	IL-4 expression was reduced in lung cells isolated from heparanase deficient mice with HDM-induced allergic inflammation	Allergic inflammation/Recruitment of eosinophils and mucus-secreting airway epithelial cells	[51]
	Administration of heparanase upregulated the expression of IL-4 in mouse immune cells	Autoimmune encephalitis/inhibition of inflammation	[200]
IL-5	IL-5 expression was reduced in lung cells isolated from heparanase deficient mice with HDM-induced allergic inflammation	Allergic inflammation/Recruitment of eosinophils and mucus-secreting airway epithelial cells	[51]
IL-6	Administration of heparanase increased IL-6 level in mouse serum	Thoracoabdominal aortic aneurysm/Systemic Inflammation	[196]
	Heparanase via its enzymatic activity upregulated IL-6 through TLR4 signaling	Inflammation	[152]
	Administration of heparanase upregulated the expression of IL-6 in mouse immune cells	Autoimmune encephalitis/Inhibition of inflammation	[200]
	Addition of heparanase enhanced the expression of IL-6 in fatty acid-stimulated macrophages	Obesity-associated breast cancer	[103]
IL-8	Heparanase enhanced IL-8 expression	Colon cancer	[183]
	Heparanase upregulated IL-8 expression via its enzymatic activity	Inflammation	[152]
IL-10	Administration of heparanase increased IL-10 level in mouse serum	Thoracoabdominal aortic aneurysm/Systemic Inflammation	[196]
	Heparanase upregulated IL-10 expression via its enzymatic activity	Inflammation	[152]
	Administration of heparanase upregulated the expression of IL-10 in mouse immune cells	Autoimmune encephalitis/Inhibition of inflammation	[200]
IL-12	Administration of heparanase downregulated the expression of IL-12 in mouse immune cells	Autoimmune encephalitis/Inhibition of inflammation	[200]
IL-17A	Silencing of heparanase resulted in a significant decrease in protein expression of IL-17A in human cervical cancer cell lines HeLa and SiHa	Promoting tumor angiogenesis, cell proliferation, and invasion in cervical cancer	[84]
iNOS	Heparanase upregulated the expression of iNOS in macrophages. Treatment of cells with the heparanase inhibitor SST0001 reduced iNOS expression	Acute kidney injury/M1 macrophage polarization	[182]
MCP-1	Addition or overexpression of heparanase upregulated the expression of MCP-1	Atherosclerosis/Macrophage activation Thoracoabdominal aortic aneurysm/Systemic	[199]
	Administration of heparanase increased MCP-1 level in mouse serum	Inflammation	[196]
	Heparanase-stimulated colon cancer cells released MCP-1	Colon cancer	[183]
	Heparanase upregulated MCP-1 via TLR4 signaling	Inflammation	[152]
	Obese heparanase knockout mice showed less MCP-1 expression compared to obese wild type mice	Obesity-associated breast cancer progression	[103]
MIP-2 (CXCL2)	MIP-2 expression was reduced in macrophages isolated from heparanase-knockout mice	Tumor/Macrophage activation and function in tumorigenesis	[153]
MMP-9	Addition or overexpression of heparanase upregulated the expression of MMP-9	Atherosclerosis/Macrophage activation	[199]
NF-κB (p65)	Knockdown of heparanase led to increased expression of nuclear NF-κB in melanoma cell lines	Melanoma progression	[169]
P21	Heparanase downregulated p21 in colon carcinoma cells through its enzymatic activity and involved TLRs and NF-κB signaling	Colon carcinoma/Modification of cell cycle	[194]
α-SMA	Protein expression of α-SMA was increased in heparanase-overexpressing mice with acute kidney injury but decreased when pre-treating the mice with heparanase inhibitor PG545	EMT/Acute kidney injury	[179]
TLR2	Heparanase knockout cells expressed less TLR2 protein	Tumor/Macrophage activation and function in tumorigenesis	[153]
TNF-α	TNF-α expression was reduced in macrophages isolated from heparanase-knockout mice	Tumor/Macrophage activation and function in tumorigenesis	[153]
	Increased expression of TNF-α in heparanase overexpressing transgenic mice with DSS-induced colitis	Ulcerative colitis/Induction of NK-κB activation	[131]
	Addition or overexpression of heparanase increased the expression of TNF-α	Atherosclerosis/Macrophage activation	[199]
	Heparanase upregulated TNF-α via TLR4 signaling. Heparanase deficiency reduced the expression of TNF-α in macrophages	Inflammation Obesity-associated breast cancer progression	[103,152]
VEGF	VEGF protein expression was decreased in heparanase knockdown cells and increased in heparanase overexpressing cells via activating the HIF1 pathway	Cervical cancer	[177]
	Heparanase overexpression led to the enhanced expression of VEGF. Heparanase regulated the expression of VEGF by mediating the activation of SRC	Tumor vascularity	[175]
	VEGF expression was increased in heparanase overexpressing melanoma cell lines and decreased in heparanase downregulated cells	Melanoma progression	[92]
Vimentin	Protein expression of vimentin was increased in heparanase-overexpressing mice with acute kidney injury but decreased when pre-treating the mice with heparanase inhibitor PG545	EMT/Acute kidney injury	[179]

EGR, early growth response; HCC, hepatocellular carcinoma; HIF, hypoxia inducible factor.

**Table 4:** Heparanase regulates protein phosphorylation.

Protein	Observation/Mechanism	Related Disease/Function	Reference
AKT	Inhibition of heparanase reduced AKT phosphorylation	Breast Cancer Brain Metastasis	[79]
	High expression of heparanase in myeloma cell lines led to increased AKT phosphorylation. This was blocked by treating cells with heparanase inhibitor SST0001	Tumor progression	[76]
Epidermal growth factor receptor (EGFR)	Inhibition of heparanase reduced EGFR phosphorylation	Breast Cancer Brain Metastasis	[79]
	Heparanase enhanced the phosphorylation level of EGFR in carcinoma cells	Tumor progression	[205]
	Heparanase released HS via shedding syndecan-1 which induced EGFR phosphorylation	Colorectal cancer	[151]
ERK	The level of phosphorylated ERK was increased in heparanase overexpressing neural stem and progenitor cells during differentiation	Promoting Embryonic stem cell differentiation into Oligodendrocytes	[207]
	Addition of exogenous heparanase induced ERK phosphorylation in macrophages	Inducing cytokine expression in macrophage	[153]
	High expression of heparanase in myeloma cell lines led to increased ERK phosphorylation. The increased phosphorylation of ERK was blocked in cells treated with heparanase inhibitor SST0001	Tumor progression	[76]
Focal-adhesion kinase (FAK)	The phosphorylation of FAK was elevated in heparanase-overexpressing breast cancer cell lines. Likewise, the phosphorylation of FAK was decreased in heparanase-knockout cell lines. Heparanase promoted cell cluster formation by regulating FAK-Src-paxillin pathway	Promotion of cell cluster formation/Tumor metastasis	[197]
IκBα/IκB	Heparanase enhanced phosphorylation of IκBα in heparanase overexpressing mice suffering colitis-associated tumors	Ulcerative colitis/Induction of NK-κB activation	[131]
	IκB phosphorylation was decreased in pancreas tissues of heparanase-overexpressing mice treated with heparanase inhibitor PG545	Acute pancreatitis	[127]
JNK	Addition of exogenous heparanase induced JNK phosphorylation in macrophages	Inducing cytokine expression in macrophage	[153]
	JNK phosphorylation was decreased in macrophages isolated from heparanase knockout mice	Tumor Growth/Induction of pro-inflammatory cytokine expression by chemotherapy-treated macrophage	[123]
MEK	Heparanase induced MEK phosphorylation via releasing HS of syndecan-1	Colorectal cancer	[151]
p38	Addition of exogenous heparanase enhanced p38 phosphorylation in macrophages	Inducing cytokine expression in macrophage	[153]
	Heparanase-overexpressing cells induced p38 phosphorylation	Promoting tumor angiogenesis	[175]
p65 NF-κB	Increased nuclear p65 phosphorylation was detected in heparanase overexpressing mice treated with DSS to induce colitis-associated tumors	Ulcerative colitis/Induction of NK-κB activation	[131]
Paxillin	The phosphorylation of paxillin was elevated in heparanase-overexpressing breast cancer cell lines. In contrast, the phosphorylation of paxillin was decreased in heparanase-knockout cell lines. Heparanase promoted cell cluster formation by regulating FAK-Src-paxillin pathway	Promotion of cell cluster formation/Tumor metastasis	[197]
SRC	The phosphorylation of SRC was increased in heparanase-overexpressing breast cancer cell lines. In contrast, the level of SRC phosphorylation was decreased in heparanase-knockout cell lines. Heparanase promoted cell cluster formation by regulating FAK-Src-paxillin pathway	Promotion of cell cluster formation/Tumor metastasis	[197]
	Inactive heparanase stimulated SRC phosphorylation	Tumor angiogenesis	[175]
	Heparanase enhanced the phosphorylation level of SRC in carcinoma cells	Tumor progression	[205]
Signal Transducer and Activator of Transcription (STAT)	Heparanase increased nuclear STAT phosphorylation	Tumor progression	[205]
STAT3	Higher number of cells positive for nuclear-localized pSTAT3 were observed in heparanase-overexpressing transgenic mice	Modulator of tumor-promoting chronic inflammation	[131]
	Heparanase enhanced STAT3 phosphorylation	Tumor progression	[205]
	Reduced STAT3 phosphorylation was observed in obese heparanase knockout mice	Obesity-associated breast cancer progression	[103]
STAT5b	Heparanase enhanced STAT5b phosphorylation	Tumor progression	[205]

VCAM-1, vascular cell adhesion molecule 1; SERPINE1, plasminogen activator inhibitor type 1; VEGFA, vascular endothelial growth factor A; FXa, activated factor X; TF, tissue factor; TGF, transforming growth factor; PDK2, pyruvate dehydrogenase kinase 2; HIF1, hypoxia inducible factor.

## Data Availability

Not applicable.
